# Obstetric and Neonatal Invasive Meningococcal Disease Caused by *Neisseria meningitidis* Serogroup W, Western Australia, Australia 

**DOI:** 10.3201/eid3002.230639

**Published:** 2024-02

**Authors:** Julie Hart, Gary K. Dowse, Michelle Porter, David J. Speers, Anthony D. Keil, Jane D. Bew, Shakeel Mowlaboccus, Charlene M. Kahler

**Affiliations:** Queen Elizabeth II Medical Centre, Nedlands, Western Australia, Australia (J. Hart, M. Porter, D.J. Speers, A.D. Keil, J.D. Bew);; Department of Health, Perth, Western Australia, Australia (G.K. Dowse);; The University of Western Australia, Perth (D.J. Speers, S. Mowlaboccus, C.M. Kahler)

**Keywords:** invasive meningococcal disease, IMD, cluster, obstetric, neonatal, Neisseria meningitidis, bacteria, serogroup W, sequence type 11, clonal complex, pregnancy, pregnant women, newborn, perinatal death, Western Australia, Australia

## Abstract

Three mother-baby pairs with invasive meningococcal disease occurred over 7 months in Western Australia, Australia, at a time when serogroup W sequence type 11 clonal complex was the predominant local strain. One mother and 2 neonates died, highlighting the role of this strain as a cause of obstetric and early neonatal death.

In Western Australia, Australia, an outbreak of serogroup W meningococcal disease in 2017 caused obstetric and neonatal cases of invasive meningococcal disease (IMD). The outbreak was caused by a hypervirulent strain of Neisseria meningitides belonging to sequence type 11 clonal complex (MenW:cc11). We report 3 cases that occurred during this outbreak. This study was approved by the Western Australia Women and Newborn Health Service Human Research Ethics Committee.

## The Study

Case 1, in June 2017, involved a 26-year-old pregnant woman (G2P1, 41 weeks) who had no concurrent conditions underwent induction with artificial rupture of membranes for fetal compromise on cardiotocography and delivered vaginally. The baby was well at birth and discharged on day 2 of life. At 5 days of age, the neonate was returned to hospital by ambulance with respiratory distress requiring intubation. Treatment with intravenous benzylpenicillin, cefotaxime, and acyclovir was given, but because of extensive hypoxic brain injury, the baby was extubated and died 2 days later. *Neisseria meningitidis* serogroup W (MenW) DNA was detected by PCR (in-house multiplex real-time PCRs for *ctrA* and *porA* genes) but was not cultured from placenta or from brain, larynx, and lung tissue at postmortem. *N. meningitidis* (not typed) was the only pathogen isolated from a maternal low vaginal swab specimen collected 1-week postdelivery to investigate vaginal discharge.

Case 2, in December 2017, involved a 36-year-old pregnant woman (G8P5, 38 weeks) who had gestational diabetes was hospitalized because of watery diarrhea and severe abdominal pain. Her condition rapidly deteriorated, and she died despite resuscitative efforts. A perimortem emergency caesarean section was performed, but the fetus was delivered without signs of life. Maternal blood culture grew MenW (isolate EXNM778, PubMLST [https://pubmlst.org/organisms/neisseria-spp] identification no. 110297), which demonstrated intermediate susceptibility to penicillin (0.25 mg/L) by Etest (bioMérieux, https://www.biomerieux.com) interpreted using Clinical and Laboratory Standards Institute (https://clsi.org) guidelines. MenW was detected by PCR from placental tissue but not from postmortem fetal blood or lung, liver, or brain tissue.

Case 3, in January 2018, involved a 22-year-old pregnant woman (G2P1, 39 weeks) who had gestational diabetes sought care in spontaneous labor with fever and fetal compromise on cardiotocography, prompting a nonelective caesarean section. The neonate had neck cord entanglement and tachypnea requiring continuous positive airway pressure. Maternal C-reactive protein (194 mg/L) and leukocyte count (34 × 10^9^ cells/L) were increased. Intravenous benzylpenicillin and gentamicin were given for suspected neonatal sepsis (C-reactive protein 21 mg/L, lactate 4.9 nmol/L). Gastric aspirate, ear, and placental swab specimens (but not blood or cerebrospinal fluid) subsequently grew MenW (isolate EXNM791, PubMLST identification no. 110300) demonstrating intermediate susceptibility to penicillin (0.25 mg/L). The neonate received intravenous cefotaxime for 5 days. Testing of whole blood and cerebrospinal fluid by PCR did not detect *N. meningitidis*. The mother initially received intravenous clindamycin, gentamicin, and metronidazole (previous rash to penicillin) and then intravenous ceftriaxone for 5 days. No growth resulted from maternal blood culture collected after antimicrobial drugs were given. Mother and baby were discharged at day 7, after rapid improvement.

**Figure 1:**
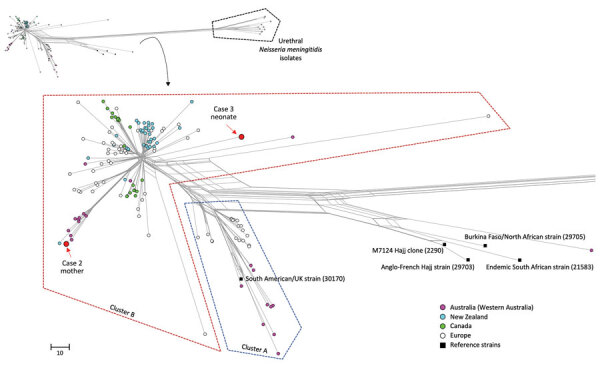
Phylogenetic relationship of 2 invasive *Neisseria meningitidis* serogroup W (MenW:cc11) strains from 3 mother-baby pairs with invasive meningococcal disease in Western Australia, Australia, compared with other local and international isolates. The neighbor-net phylogenetic network was constructed based on 1,605 core genome loci using the Genome Comparator tool available on the PubMLST *Neisseria* website (https://pubmlst.org/organisms/neisseria-spp). Red indicates the 2 isolates reported in this study ; pink circles indicate isolates from Western Australia reported by Mowlaboccus et al. (*1*); blue, green, and white circles indicate isolates from the Australian Penicillin Resistance–Associated Lineage reported by Willerton et al. (*3*) from New Zealand, Canada, and Europe, respectively; black squares indicate reference invasive MenW/cc11 strains characterized by Lucidarme et al. (*4*). Identification numbers in parentheses indicate PubMLST identification numbers of reference isolates. Inset at top shows full phylogenetic trees; callout at left shows urethral *N. meningitidis* strains strains from Tzeng et al. (*2*) and Ma et al. (*5*), which were isolated from urethral swabs in the United States (NM1, NM2), the United Kingdom (M11_240294, M11_241043, M13_240559), Italy (PE5, PE6, PE7), and France (LNP26948, LNP27256). Scale bar indicates number of different loci among the 1,605 compared.

We performed typing of case isolates by nested PCR directed at the *porA* gene covering 2 variable regions (VR1 and VR2), followed by gel electrophoresis and Sanger sequencing analysis, with comparison to sequences in the PubMLST database. Whole-genome sequencing (*1*) of case 2 and 3 isolates demonstrated phylogenetic clustering in cluster B (penicillin resistance–associated lineage) of MenW:cc11 lineage 11.1, which is distinct from lineage 11.2, to which the US nongroupable urethritis strain belongs (*2*) (Figure 1). Only case 2 clustered with isolates from other local contemporaneous cases, part of an outbreak that began in 2014 (*1*).

## Conclusions

**Figure 2:**
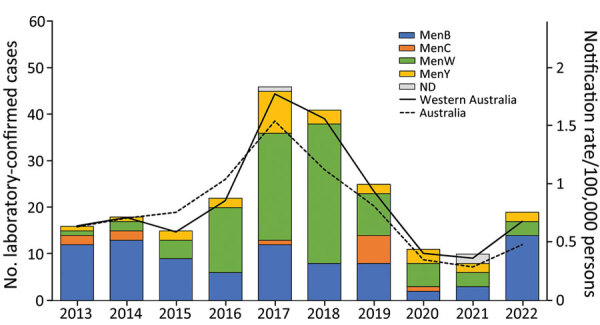
Notifications of laboratory-confirmed invasive meningococcal disease cases in Western Australia and Australia, by serogroup and year of onset, 2013–2022. Serogroup data were extracted from the Western Australian Notifiable Infectious Diseases Database (https://www.health.wa.gov.au), and the overall national notification data were obtained from the National Notifiable Diseases Surveillance System (https://www.health.gov.au/our-work/nndss). Rates were calculated by using estimated resident population data from the Australian Bureau of Statistics (https://www.abs.gov.au) (national, state and territory population, March 2023, accessed on October 3, 2023). Men, meningococcal; ND, not determined.

The recent rapid global expansion of hypervirulent MenW:cc11, which emerged in the late 1990s from South America and spread to Europe, North America, and Australasia (*3*), caused a rapid increase in IMD incidence in Western Australia, from an average of <1 MenW case/year before 2014 to 30 cases among a population of 2.6 million persons in 2018 (Figure 2). Some MenW:cc11 strains have been associated with a high case-fatality rate and atypical disease manifestations (*3*).

Isolates from case 2 (mother) and case 3 (neonate) belonged to the UK/South America MenW:cc11 lineage (*3*) and to the penicillin resistance–associated lineage previously described from Western Australia (*1*), which has since expanded to 8 countries (*3*). Those isolates have demonstrated intermediate penicillin resistance by Clinical and Laboratory Standards Institute guidelines and have been associated with treatment failure using low-dose penicillin regimens recommended for IMD. The 2 isolates we describe were not closely related to lineage 11.2 urethritis isolates and did not possess previously described adaptations to urogenital infection; they had intact capsule genes and did not harbor the gonococcal *aniA* and *norB* alleles that promote urethral anaerobic growth (*2*,*5*). Our cases are most likely to represent atypical disease manifestations of MenW:cc11 resulting from ascending maternal genital infection (case 1) or from maternal IMD (case 2).

*N. meningitidis* is primarily spread by the respiratory route, but genital meningococcal disease is reported (*5*). Although a retrospective matched case–control study (*6*) has shown a strong association between childhood IMD and coincident pregnancy of the patient’s mother, increased meningococcal carriage rates in pregnant women have not been shown, and few case reports of IMD during pregnancy have been published (*7*).

National surveillance data from England (2011–2014) (*7*) included 4 cases in pregnant women but indicated that pregnant women were 5 times less likely to have IMD develop than were nonpregnant women. This finding highlights the unusual occurrence of 3 mother-baby pairs within 7 months in a much smaller population. Additional reported cases in pregnancy include 5 with meningitis and single cases with acute meningococcemia, chronic meningococcemia, and pericarditis (*8**–**12*). In total, those 12 cases in pregnant women caused 4 neonatal deaths. The England surveillance study also identified 5 cases of early-onset (<7 days) neonatal IMD, none with reported maternal illness (*7*). Fetal or early-onset neonatal IMD can occur after colonization of the maternal genital tract or by septicemic transmission in utero from maternal IMD (*13*). Early neonatal IMD is rare, presumably because of the rarity of genital tract colonization plus maternal antibody transfer across the placenta (*13*).

A review in 2017 described 23 cases of early-onset (<8 days) neonatal IMD with a high case-fatality rate (34.8%) (*14*). A 2020 review (*13*) and 2 case reports (*15*) added an additional 8 cases, with 2 neonatal deaths. In total, those 31 cases resulted in 10 neonatal deaths, although the 3 known MenW case-patients survived.

MenW incidence in Western Australia has decreased substantially, from 30 cases in 2018 to 3 cases in 2021 (Figure 2). This decrease is probably associated with MenACWY vaccination programs for adolescents 15–19 years of age and children 12 months–4 years of age, which began in Western Australia in May 2017 and January 2018, respectively, along with the effect of subsequent COVID-19–related public health restrictions.

In summary, we report a time-place cluster of 3 mother-baby pairs with *N. meningitidis* infection, ranging from vaginitis to fulminant fatal maternal and neonatal sepsis, to highlight the potential for MenW:cc11 to cause obstetric and early neonatal infection. Because *N. meningitidis* is uncommonly isolated from the genital tract and does not necessarily cause disease, there is no current recommendation to routinely screen or treat pregnant women. However, these cases suggest the need for opportune laboratory reporting of *N. meningitidis* isolates from the genital tract; if pregnant women are found colonized, preemptive treatment should be considered to prevent subsequent neonatal infection. Our findings also highlight the need for MenACWY vaccination of adolescents and possible opportunistic catch-up vaccination in women before or during pregnancy.
